# Grape Pomace Polyphenols as a Source of Compounds for Management of Oxidative Stress and Inflammation—A Possible Alternative for Non-Steroidal Anti-Inflammatory Drugs?

**DOI:** 10.3390/molecules27206826

**Published:** 2022-10-12

**Authors:** Veronica Sanda Chedea, Ștefan Octavian Macovei, Ioana Corina Bocsan, Dan Claudiu Măgureanu, Antonia Mihaela Levai, Anca Dana Buzoianu, Raluca Maria Pop

**Affiliations:** 1 Research Department, Research Station for Viticulture and Enology Blaj (SCDVV Blaj), 515400 Blaj, Romania; 2 Faculty of Medicine, “Iuliu Hatieganu” University of Medicine and Pharmacy, 400012 Cluj-Napoca, Romania; 3 Department of Pharmacology, Toxicology and Clinical Pharmacology, Faculty of Medicine, “Iuliu Hatieganu” University of Medicine and Pharmacy, No. 23, Marinescu Street, 400012 Cluj Napoca, Romania; 4 Department Mother and Child, Faculty of Medicine, “Iuliu Hatieganu” University of Medicine and Pharmacy, No. 3–5, Clinicilor Street, 400012 Cluj Napoca, Romania

**Keywords:** grape pomace, polyphenols, antioxidant, anti-inflammatory

## Abstract

Flavonoids, stilbenes, lignans, and phenolic acids, classes of polyphenols found in grape pomace (GP), were investigated as an important alternative source for active substances that could be used in the management of oxidative stress and inflammation. The benefic antioxidant and anti-inflammatory actions of GP are presented in the literature, but they are derived from a large variety of experimental *in vitro* and *in vivo* settings. In these *in vitro* works, the decrease in reactive oxygen species, malondialdehyde, and thiobarbituric acid reactive substances levels and the increase in glutathione levels show the antioxidant effects. The inhibition of nuclear factor kappa B and prostaglandin E2 inflammatory pathways and the decrease of some inflammatory markers such as interleukin-8 (IL-8) demonstrate the anti-inflammatory actions of GP polyphenols. The *in vivo* studies further confirmed the antioxidant (increase in catalase, superoxide dismutase and glutathione peroxidase levels and a stimulation of endothelial nitric oxide synthase -eNOS gene expression) and anti-inflammatory (inhibition of IL-1𝛼, IL-1β, IL-6, interferon-𝛾, TNF-α and C-reactive protein release) activities. Grape pomace as a whole extract, but also different individual polyphenols that are contained in GP can modulate the endogenous pathway responsible in reducing oxidative stress and chronic inflammation. The present review analyzed the effects of GP in oxidative stress and inflammation, suggesting that it could become a valuable therapeutic candidate capable to reduce the aforementioned pathological processes. Grape pomace extract could become an adjuvant treatment in the attempt to reduce the side effects of the classical anti-inflammatory medication like non-steroidal anti-inflammatory drugs (NSAIDs).

## 1. Introduction

The study of plant-derived compounds received increased attention among researchers worldwide in the attempt to discover new bioactive molecules with marked therapeutic actions and minimal adverse effects. A healthy diet and regular exercise protect the human body from cardiovascular problems, diabetes, and cancer [1]. The definition of a healthy diet is permanently evolving and changing to comprise all the knowledge with respect to different foods, nutrients or food components in health and disease. Thus, a healthy food approach refers to diets rich in plant-based foods (fresh fruits, vegetables, legumes, seeds, whole grains, nuts) and low in animal-based products (e.g., fatty and processed meats) [2]. Therefore, WHO recommends consuming 400 g (5 portions) of fruit and vegetables to our diet [3] because they possess significant levels of phytochemicals like flavonoids, stilbenes, lignans, and phenolic acids found in different concentrations [1,4,5].

Grapes, with a global production of around 78 million tons in 2020 [6], and with around 75% of total production going into the wine industry, are such an example. One of the main reasons for grapes research is the quantity of waste resulting from the juice and winemaking process [7,8,9]. An important by-product of the winemaking industry is grape pomace (GP), and due to the important consumption of wine (27 million tons in 2019) [6], there are also important quantities of GP generation (8.49 million tons) [10]. Disposal of this by-product causes an ecological problem because of pollution and other hazardous issues that come with producing large quantities in a short time and little space for deposit [11]. To overcome these, different solutions were proposed for by-products valorization, including the recovery of containing bioactive compounds like phenolic compounds [12,13,14]. Polyphenols have beneficial effects on various diseases, especially because of their anti-inflammatory and antioxidant effects via different mechanisms [15,16,17]. Accordingly, it was observed that GP, because of its various and rich quantity of phenolics has potential antioxidant, anti-inflammatory, anti-microbial, anti-cancer effects and also beneficial cardiovascular and hepatic effects [18,19,20,21]. Knowing that oxidative stress and inflammation processes are common to many diseases, this review will focus especially on these pathophysiological processes since their management is a key step in the attempt to prevent or reduce disease progression. Thus, their modulation by polyphenols from GP as reported in both *in vitro* and *in vivo* studies will be addressed. Also, another important aim of this review is to compare the anti-inflammatory effects of GP polyphenols with the one of non-steroidal anti-inflammatory drugs (NSAIDs). Non-steroidal anti-inflammatory drugs are used for medical purposes since ancient times, being one of the most prescribed drug classes in the world. They possess anti-inflammatory, antipyretic, and analgesic effects and therefore are prescribed for many diseases, especially chronic diseases like rheumatoid arthritis, osteoarthritis, ankylosing spondylitis, and chronic pain [22,23,24]. Unfortunately, the therapeutic effects can be associated with pulmonary, renal, gastrointestinal, cardiovascular, and hepatic adverse reactions that might lead to severe complications [25,26,27,28]. Thus, there is an urgent need in identifying novel antioxidant and anti-inflammatory agents, therapeutically potent and with minimal side effects that could be used instead of classical drugs or as add-on therapy, and GP could be a source of such kind of compounds.

## 2. Oxidative Stress Process

An imbalance between the synthesis and accumulation of oxygen reactive species (ROS) in cells and tissues and the capacity of an organism to eliminate these reactive compounds results in oxidative stress [29]. Thus, organism homeostasis can be altered if higher accumulation of free radicals occurs. ROS are normally generated through different reactions like enzymatic and non-enzymatic, and they can have exogenous or endogenous sources. The enzymatic reactions responsible for ROS generation are characterized by phagocytosis, cytochrome P450 reactions, mitochondrial respiratory chain, and cyclooxygenase-synthesis of prostaglandin [30]. The non-enzymatic system involves the reaction of free oxygen with organic molecules or through tissue-radiation exposure [29]. The exogenous sources of free radicals production are represented by radiation [30], air pollution, and cigarette smoke while endogenous sources are represented by the enzymes systems: mitochondrial respiratory chain, nicotinamide adenine dinucleotide phosphate (NADPH) oxidase family, cyclooxygenase, and lipoxygenase [31,32,33,34]. 

ROS production direct measurement is a very complex and difficult process mostly because of ROS sources and species variety, low steady-state concentrations, high reactivity, and of the detection methods involved in the measurement analysis. Also, the oxidative stress assessment is performed by the indirect evaluation of ROS-induced damage on biological targets like DNA, proteins, membrane lipids, and others [35]. Additionally, ROS generation differs significantly in tissue localization, activation mechanisms, and functions in diseases. As a result, ROS levels must be kept within a range that enables organisms to function normally. These concentration ranges may vary between tissues. However, the balance between physiological redox states and oxidative stress is fragile and is based on relative rates of ROS production and destruction. All of these make it difficult to establish an absolute scale that can offer the values of ROS concentrations in physiological and pathological conditions [36]. Usually, ROS in low and moderate concentrations doesn’t possess a significant threat to homeostasis, their beneficial role being known in different physiological processes (e.g., the synthesis of different cellular structures, the help of defense system to neutralize pathogen agents) [29]. If the free radicals are present in large quantities, they cross the physiological barrier and can lead to several health issues, such as cardiovascular, neurological, kidney, respiratory, and rheumatoid diseases [32,34,37]. To counter the side effects of ROS, the organism possess antioxidant mechanisms represented by enzymatic systems (superoxide dismutase—SOD, catalase—CAT, glutathione peroxidase—GPx) and non-enzymatic ones (glutathione, vitamins A, C, and E) [32,33,37]. Superoxide dismutase (SOD) is responsible for O_2_^−^ reduction to H_2_O_2_. Also, another role of this enzyme is to prevent the formation of other free radicals (peroxynitrite—ONOO^−^). Further, the H_2_O_2_ molecule will be converted by catalase (CAT) in water and oxygen [33,38]. Glutathione peroxidase (GPx) is another enzyme with a role in degrading H_2_O_2_ and hydroperoxide molecules using glutathione (GSH) as a proton donor [32]. 

Another category of endogenous substances resulting from internal metabolism with consequences on human health due to an imbalance between synthesis and elimination is reactive nitrogen species (RNS). RNS are already known to take part in the pathophysiological process of different diseases (diabetes, Parkinson’s disease, pulmonary, cardiovascular, rheumatological, liver, and neurological diseases) [39]. RNS are synthesized through the interaction of nitric oxide molecules with other reactive oxygen species. Nitric oxide (NO) is a versatile natural molecule found in organisms resulting from the breakdown of arginine to citrulline [40]. Nitric oxide possesses antimicrobial activity, promotes vascular relaxation with reducing blood pressure, and cell signaling. NO also acts as a scavenger molecule interacting with superoxide anion (O_2_^−^) leading to peroxynitrite (ONOO^−^) formation. ONOO^−^ leads to endothelial damage, DNA oxidation, and lipid peroxidation [40,41]. Nitrogen dioxide (NO_2_^−^) is another RNS, that results from the interaction of NO with peroxyl radical, which produces lipid peroxidation, ascorbic acid oxidation, and alters tyrosine structure and function leading to the 3-nitrotyrosine formation (3NT) [40,42]. 

Even though the role of ROS and RNS in damaging the cells and signal transduction is well known, there are still several highly debated issues that need to be resolved. When present in low quantities, ROS and RNS operate as regulatory mediators in signaling processes; nevertheless, when present in high concentrations, they are toxic to living organisms by inactivating critical cellular components. This indicates that the concentrations of ROS and RNS control the change from their favorable to unfavorable effects, although the concentrations at which this change occurs are not well known [36].

The imbalance between the increased ROS and RNS production and decrease of antioxidant molecules will eventually lead to chronic inflammation [43]. During the oxidative stress process, the reactive oxygen/nitrogen species can initiate intracellular signaling and through which specific proinflammatory genes are expressed [43,44]. Generally, between oxidative stress and inflammation, there is a state of interdependency, with one potentiating the other via different mechanisms. For example, during oxidative stress at the brain level, lipids and proteins suffer alterations through oxidation which conduct to disruptions in neurons’ communication with inflammation being stimulated [45]. During oxidative stress, DNA suffers damage resulting in different metabolites (8-oxo-7 8-dihydro-2′-deoxyguanosine, 8 oxo-guanine). It was demonstrated that 8-oxo-guanine presence stimulates the nuclear factor kappa B (Nf-κB) [46]. The activation of Nf-κB enhances the pro-inflammatory response through increased synthesis of inflammatory molecules (cytokines, chemokines) and also activation of immune cells [47,48,49]. Cytokines manage also to increase the ROS levels in non-immune cells. For example, it was shown that IL-6 stimulates the NADPH in lung cancer cell lines leading to an increase in ROS levels [47]. Oxidative stress leads to the release of arachidonic acid which under cyclooxygenase and lipoxygenase enzymes reactions, results in prostaglandins and leukotrienes synthesis [49]. Also, oxidative stress can be enhanced by inflammation. During inflammation, immune cells responsible for phagocytosis (neutrophils and macrophages) produce reactive oxygen and nitrogen species to dissolve the pathogen. In the case of an exaggerated inflammatory response, these reactive species exit from phagocytic cells resulting in tissue injury outside the inflammatory site [47].

Thus, inflammation and oxidative stress, are tightly interconnected [50] initiation and maintenance of many pathological conditions, their prevention and control could provide a safe alternative in chronic disease management.

## 3. Inflammation

Inflammation represents a pathophysiological mechanism of defense that acts in case of homeostasis perturbations provoked by infectious agents or trauma. Immune cells enact this mechanism which has a role in locating the pathological agent, digestion, and resolution of the inflammation with restoring homeostasis. Thus, inflammation could be considered a protective response, but an uncontrolled inflammation may be potentially harmful and may lead to many acute and chronic diseases [51].

Inflammation could be acute or chronic depending on the interval of time from the onset of homeostasis impairment until the development of the entire process and the appearance of clinical symptoms. Acute inflammatory diseases present a rapid and non-specific immune response which usually lasts up to 2 weeks with the resolution of the inflammation process [52]. If the process is not healed, this can lead to a prolonged immune response called chronic inflammation, which lasts from months to years [53]. Thus, chronic inflammations may require a long-lasting management and they become a burden not only for individuals but also for society due to higher costs and health assistance. For all these reasons, there is a perpetual race to discover new molecules with pharmacological properties that may limit the chronic evolution of inflammation. 

The etiology of it is also variable, different agents like infectious agents or trauma could trigger cells belonging to both innate and adaptive immunity. The most important aspect of acute inflammation is the recognition of the pathogen or damaged tissue through circulating molecules, which signals innate immune cells and leads to a cascade of biological reactions [54,55]. These reactions are mediated by several proinflammatory molecules, such as cytokines and chemokines, which act interconnected in a signaling network, leading to the recruitment of more immune cells and mediators at the site of inflammation [56,57]. Vasodilation also occurs at the site of inflammation to bring the immune cells there. This process is facilitated by local mediators (nitric oxide, prostaglandins) produced by endothelial and inflammatory cells, leading to the translocation of vascular fluid into interstitial space and enhancing the migration of immune cells [58,59].

Neutrophils represent the first and the most important type of cells belonging to innate immunity that act at the site of inflammation. Neutrophils create a toxic environment by releasing cytotoxic compounds from their vesicles, such as proteases and reactive oxygen and nitrogen species, that destroy the pathogen and the surrounding tissue [55,60]. After the pathogen is destroyed, the resolution process begins, and monocytes are recruited for wound healing. Monocytes block other new inflammatory processes with possible downside effects for the host [55].

Clinical signs and symptoms that characterize inflammation reflect these pathophysiological processes. Locally at the inflammation site, heat, edema, redness, pain, and impaired function could be observed [57]. Cellular injury inducing an immune response activates the intrinsic blood coagulation pathway [61]. Coagulation is activated to create the fibrin clot, which has a role in the isolation of the inflammation process, but also it enhances the pro-inflammatory response [62]. Another mechanism that intervenes in innate immunity is the complement system, which consists in several proteins that could be activated. These proteins, through a series of chain reactions, create a so-called “membrane complex attack” that disrupts the microbe’s cell membrane and induces death [58,59,62]. 

The acute inflammatory response may transit to a chronic response if the neutrophils fail to eliminate the pathogen agent in the first place. After this, the innate immune response is followed by an adaptive immune response characterized by the presence of macrophages and lymphocytes. Besides them, fibroblasts and plasma cells can be found at the site of chronic inflammation [63]. Monocytes are a group of cells that migrate from blood to different tissues and differentiate themselves into macrophages. Macrophages work in innate immune response alongside neutrophils and in the adaptive immune response through activating lymphocytes [59,63]. Lymphocytes T (Ly T) are activated by macrophages and will be divided into different populations with specific role in the adaptive immune response. However, their primary function is to enhance the immune system, stimulating all immune cells to give a specific defense response against the etiological agent [63]. Lymphocytes B (Ly B), activated by Ly T cells, differentiate in plasma cells which produce antibodies against different types of antigens [64]. The chronic process is associated with tissue destruction, and the repair process is maintained by fibroblasts that secrete collagen, representing the main component needed for wound healing [65]. Thus, acute inflammation is characterized by vasodilatation, and innate immune cells, while the chronic one is described by the involvement of adaptive immune cells, and fibroblast proliferation with significant changes in wound healing. Chronic inflammation is an important component of many diseases, such as atherosclerosis, diabetes, metabolic disorders, cancer, and autoimmune conditions, so the conversion of acute inflammation to a chronic one is a desirable outcome.

## 4. Grape Pomace Generation

Historically, the first evidence of wine production was found in south Caucasus and dated back as far as 6000 BC. From there, traces of wine production were discovered in Syria, Palestine, and Egypt. It spread around the Mediterranean basin during Roman times and further in Europe as long as the Roman Empire was further invading [20,66].

In European Union, as of 2020, 3.2 million hectares were used for grapevine growing and grape production, about 45% of total global grapevine growing surfaces [67]. Most of the grapes produced are sent to the wine industry, which leads to large quantities of waste products, such as GP. Grape pomace is generated through the vinification process which comprises all the technological phases from grape harvesting to wine bottling. Destemming is the first process after harvesting grapes, removing stems and grape stalks. After this, crushing takes place in which grapes are mashed into juice, skins, pulp, and seeds [66]. Maceration follows when juice (must) remains alongside grape skins and pulp seeds for different periods depending on what type of wine is produced. In the case of aromatic white wine, the maceration takes a shorter time, while for red wine it will be longer [20,66].Afterwards, in the pressing process, the must is extracted from grape waste. Therefore, there are two types of GP, red and white, according to the grapes used in the vinification process. The main difference between red and white wine is that white must is separated from pomace before fermentation. In contrast, in the case of red wine, pressing happens only after fermentation. Also, while pressing, the wine will gain its color and flavor [12,20,68]. After the pressing process, where must (red or white) is extracted, the remaining GP has different properties. For example, in terms of pressing and fermentation, white GP is sweet due to its higher sugar content because it didn’t undergo fermentation. At the same time, red GP is a fermented matrix containing different concentrations of alcohol [20]. At the same time, the polyphenols content varies depending on the vinification technology, respectively red or white. 

### Grape Pomace Polyphenols

**Figure 1:**
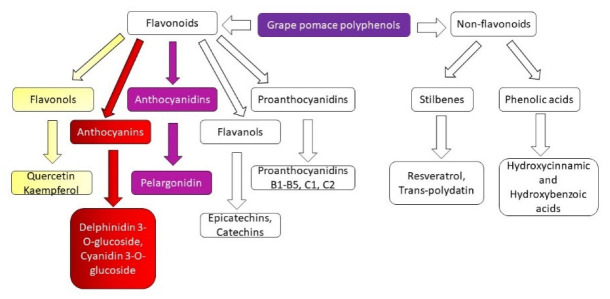
Grape pomace polyphenols classes.

From the chemical structure point of view, polyphenols are compounds made from several hydroxyl groups attached to an aromatic ring. These molecules can vary from simple to complex structures [69]. Also, these compounds are synthesized in plants and exist as glycosides, which are further formed from the basic polyphenol structure and glycosylic radical (sugar fragment) [70]. GP polyphenols are divided into two classes: flavonoids and non-flavonoids (Figure 1). Grape pomace flavonoids are further subdivided into flavonols, anthocyanins, flavanols, proanthocyanidins, and anthocyanidins, while GP non-flavonoids in stilbenes and phenolic acids [71,72]. 

**Figure 2:**
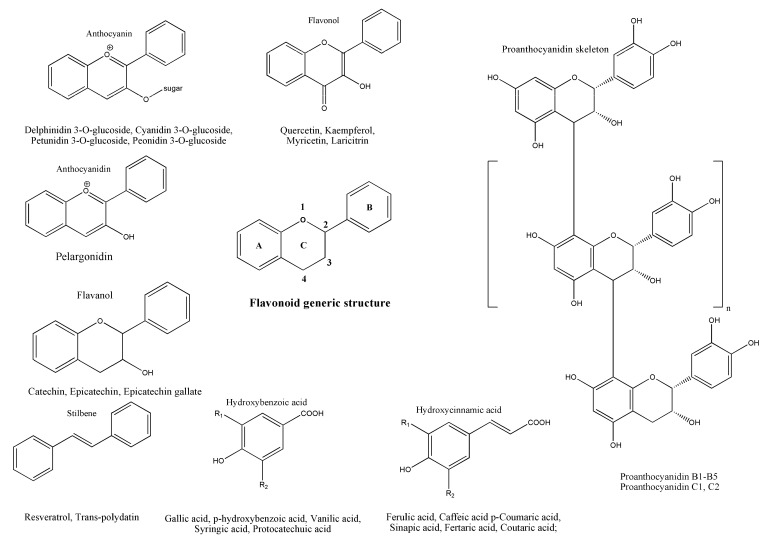
Flavonoid generic structure, structures of the main grape pomace polyphenols classes, as well as the most representative compounds.

The main structure of a flavonoid is made up of two phenyl radicals (rings A and B) linked to a heterocyclic ring, which contains an atom of oxygen (ring C). Based on the oxidation state and hydroxyl radicals’ distribution pattern on the heterocyclic ring, flavonoids are further divided into the classes mentioned before [69,70] (Figure 2).

Flavonols structure presents a C2−C3 double bond with a hydroxyl radical at the C3 position and ring B coupled to the C2 position. Flavanols structure presents with a hydroxyl radical situated at the C3 position, and their ring B is attached to the C2 position [70,73,74] (Figure 2). Proanthocyanidins, also called procyanidins or condensed tannins, are formed from subunits of flavanols that bond together in dimers (usually referred to as B-series—B1, B2, B3, B4, and B5) or trimers (known as C-series—C1, and C2). This series of procyanidins are being found in grape skins and seeds [75] (Figure 2). Anthocyanins present double bonds in the heterocyclic ring, their aromatic ring B being bonded to the C2 position. They represent the glycosylated form of anthocyanidins (aglycone) which result from the bond between the hydroxyl group at C3 and the sugar fragment and are the most abundant polyphenols in the peel of red grapes [70,74] (Figure 2). It is known that anthocyanins are only found in red GP because they act as a natural colorant, giving the red grape specific color. High contents of anthocyanins are found in red GP also because of the red grape skin thickness as compared to the white ones [76,77].

Compared to flavonoids, non-flavonoid polyphenols have one aromatic ring as a basic structure. Non-flavonoid molecules found in GP are phenolic acids, and stilbenes [69,70,78]. Phenolic acids are further divided into hydroxybenzoic and hydroxycinnamic acids. As representants of hydroxybenzoic acids, there are gallic, p-hydroxybenzoic, and syringic acids, and for the hydroxycinnamic acids, caffeic, p-coumaric, ferulic and synaptic acids are the most found in GP [69,70,78,79]. Stilbenes are formed from two aromatic rings bounded through the ethylene radical. The most known and studied stilbene is resveratrol. Besides GP, stilbenes are reported to be found also in grapes, and wine [69,70,78] (Figure 2). 

The polyphenolic composition from different assortments of GP may differ based on the grape cultivar, type of soil, weather, geographical location, and winemaking process [76,80]. The content of polyphenols found in GP differ from study to study; some suggest that red GP possess the highest content of polyphenols, and other suggest otherwise, but the principal idea is that no matter which GP is analyzed, all of them possess high quantities of polyphenols. For instance, Kammerer et al. 2004 studied the polyphenol composition of 14 different red and white GP [79]. The study did not find significant differences in composition between red and white varieties except for the presence of anthocyanins found in red ones [79].

## 5. Grape Pomace Polyphenols Benefic Actions 

**Figure 3:**
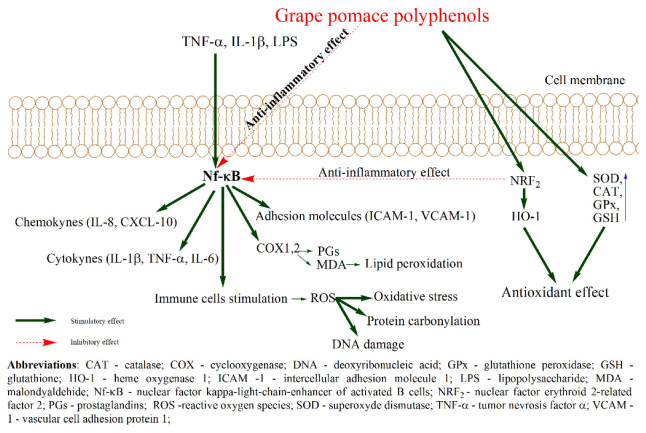
Proposed antioxidant and anti-inflammatory actions of polyphenols from grape pomace.

Grape pomace polyphenols research studies have grown in the last decades, given their potential benefic effects on promoting human health. Some of their benefic actions are observed in oxidative stress and inflammation aiming at homeostasis restoration. Regarding the antioxidant effect, polyphenols can modulate the endogenous pathway responsible for combating oxidative stress. These effects can be achieved by polyphenols capacity to activate the nuclear factor E2 and to up-regulate superoxide dismutase, catalase, glutathione, glutathione peroxidase, and heme-oxidase 1 [81,82] or their capacity to scavenge and chelate reactive oxygen species involved in ROS production [83] (Figure 3). In inflammation, polyphenols are reported to inhibit the mitogen-activated kinase pathway, Nf-kB, anddown-regulate cytokines and chemokines [81,82]. Polyphenols also inhibit cyclooxygenase and lipoxygenase, which are involved in the arachidonic acid signaling pathway, being responsible for synthesizing prostaglandin, thromboxane A2, and leukotrienes which further increase inflammatory response [73,74,82] (Figure 3).

Further, there are presented the *in vitro* and *in vivo* beneficial effects of different red and white GP in oxidative stress and inflammatory conditions.

### 5.1. In Vitro Beneficial Actions of Grape Pomace in Oxidative Stress and Inflammation

The *in vitro* studies, as presented in Table 1, can offer the possibility to investigate and identify the diversity of related diseases in which GP exerts the optimum antioxidant and anti-inflammatory effects. 

The *in vitro* beneficial action of GP was studied by Goutzourelas et al. (2015) [84]. They investigated an extract of red GP on muscle and endothelial cells using non-cytotoxic doses to check the effect of GP polyphenols extract on cells’ antioxidant enzymes [84]. The red GP extract was investigated as a mixture of compounds that contained phenolic acids (caftaric acid, gallic acid), anthocyanins, flavanols (epicatechin and catechin), flavonols (quercetin), and anthocyanidins. It was observed that GP treatment increased Glutathione S-transferase (GST) and GSH levels in both cell lines. CAT levels were decreased in endothelial cells, while in muscle cells it showed no significant differences. SOD and HO-1 presented no differences in any population. An explanation for these inconstant findings, in which some of the antioxidant enzymes are not modified, is the ability of GP to enhance other antioxidant systems (GSC, GSH) [84] (Table 1). Another *in vitro* study, of Pop et al. (2022), investigated the antioxidant effect of red GP (mixture of Pinot Noir, Cabernet Sauvignon, Fetească Neagră, and Mamaia cultivars) and white GP (mixture of Sauvignon Blanc and Muscat Ottonel cultivars) added to a mouthwash on both H_2_O_2_ exposed and non-exposed fibroblast cells [90]. They observed that both red grape pomace (RGP) and white grape pomace (WGP) decreased ROS levels in a dose-dependent matter (100 < 200 < 300 µg/mL). Similar to the non-exposed condition, in the presence of H_2_O_2_, red GP and white GP led to a significant decrease in ROS levels, the only difference being that while red GP effect was dose-dependent, and white GP produced a non-dependent action [90]. Moreover, they also studied the anti-inflammatory effects of these extracts on lipopolysaccharides (LPS) induced inflammation in cells [90]. It was observed that while in the case of white GP a dose of 100 µg/mL was sufficient to induce a significant reduction of interleukin (IL) -8 levels, for red GP was necessary a higher dose of 200 µg/mL. At the dose of 300 µg/mL, both extracts significantly reduced IL-8 levels, but not even the highest dose did significantly reduce the levels of IL-6. In the case of IL-1β, the lowest dose, 100 µg/mL, reduced its level to a similar one found in the non-exposed cells, while the doses of 200 and 300 µg/mL reduced, even more, the levels of IL-1β [90].

Marzulli et al. (2018), treated mononuclear cells with phorbol 12-myristate 13-acetate (PMA) to activate inflammation, and with different GPs (red Negroamaro cultivar or white Koshu cultivar) extracts (water, ethanol), to observe their immunomodulatory effects [91]. In terms of cytokine release, all GP fractions and extracts increased anti-inflammatory (IL-10) and pro-inflammatory (IL-12, IL-1β, IL-6, tumor necrosis factor-alpha (TNF-α)) cytokines. The water extracts of both GPs managed to increase T regulatory cells and forkhead box P3 (FoxP3) protein, which is responsible for the genes activity control that are involved in the immune system regulation. Another benefic effect of GPs extracts is FoxP3 increase which is a marker with a role in stabilizing the T regulatory cells’ function. All extracts lowered the release of granzyme (GrB) compared to PMA treated group [91]. GrB is an enzyme secreted by cytolytic T cells with role in cell necrosis leading to harmful effects on homeostasis [91]. Regarding intracellular cytokines, the water extract of red Negroamaro GP increased TNF-α and IL-10 content in monocytes, while the red Negroamaro GP ethanol extract increased IL-12 and IL-10 levels in lymphocytes. Further, the white Koshu GP water extract increased monocyte levels of IL-10 and IL-12, while the white Koshu GP ethanol extract increased lymphocyte levels of TNF-α and IL-10. IL-10 was increased by both water or ethanolic, red or white GP extracts and as underlined by authors [91], the release of IL-10 by T cells and monocytes is a key step in maintaining the immune homeostasis. In conclusion, GPs extracts could induce immune homeostasis through the anti-inflammatory IL-10 secretion which counterbalances the pro-inflammatory cytokines (IL-12 and TNF-α) [91]. Another study that reinforces the anti-inflammatory effects of RGP from *Vitis vinifera* L. cv. Montepulciano d’Abruzzo on LPS-stimulated macrophages is that of Mollica et al. (2021). They observed that the extract significantly inhibited the release of cytokines (IL-6, TNF-α, and IL-1β), the maximal inhibitory action being at the dose of 100 μg/mL [92].

The possible potential impact of GP extracts on *in vitro* calcitonin gene-related peptide (CGRP) secretion was investigated as a potential mechanism to influence migraine [85]. The treatment of CA 77 cells with different red GP extracts showed a significant decrease in CGRP levels. CGRP is a gene that represents a key mediator of migraine-induced inflammation [85]. The results suggest that GP extracts had anti-inflammatory effect preventing the release of CGRP in migraine [8885].

White GP extract and a mixture of red and white GP extract, in different concentrations (100, 200, 500 μg/mL dry extract *w*/*v*), were added to Caco-2 cells after treatment with an inflammation inducer (IL-1β) to observe the effects on IL-8 secretion and NF-κB expression [93]. Grape pomaces were hydrolyzed enzymatically to determine if anti-inflammatory effects would be augmented. Both white and red GP contained quercetin, catechin, resveratrol, gallic and caffeic acids, trans-resveratrol, rutin, and procyanidin B2 [93]. All GP fractions (100, 200 μg/mL dry extract *w*/*v*) with or without enzymatic transformation decreased ROS levels, while treatment with GP extracts in higher concentration (500 μg/mL dry extract *w*/*v*) showed a considerable increase in ROS levels. Furthermore, NF-κB expression and prostaglandin E2 (PGE2) levels were significantly reduced in all fractions. At the same time, IL-8 secretion revealed a more substantial drop in enzymatically treated fractions of mixed GP, presenting beneficial effects of enzyme hydrolysis. The mixed GP had a more potent anti-inflammatory effect due to the high content of anthocyanins found in red GP [89].

Concerning the benefic antioxidant and anti-inflammatory GP actions, the literature presents a large variety of experimental settings that can be considered for future *in vivo* research. Also, we can observe that there is still space for other hypotheses, for both red and white GPs, but especially for the white ones which were much less investigated.

Further, in the next step the GP effects *in vivo* studies were analyzed. The *in vivo* studies usually use rodents to induce different models of inflammation, but fish and lamb were also introduced.

### 5.2. In Vivo Beneficial Actions of Grape Pomace in Oxidative Stress and Inflammation

The effect of GP extracts on the pathophysiology of oxidative stress and inflammation in various types of diseases can be well documented using different *in vivo* experimental models. These types of studies are very important in deciding whether the GP can be further used in safe conditions in human clinical trials. 

The antioxidant and anti-inflammatory effects of both fresh and fermented GP extracts (*Vitis vinifera* L. cultivars, Fetească neagră, and Pinot noir, from Romania) were investigated using and a rat model of induced inflammation by turpentine oil [94]. The administration of turpentine oil increased the total oxidative status, oxidative stress index and reduced total antioxidant reactivity [94]. Treatment with GP decreased total oxidative status and oxidative stress index in a dose-dependent manner, but total antioxidant reactivity was not modified. All GP’s fractions significantly reduced malondialdehyde (MDA) levels. Total thiols were considerably lessened by turpentine, but GP managed to increase them in a concentration-dependent way. The same results were observed in the case of NOx production. 3NT was also increased by turpentine, but GP varieties decreased the levels. Due to higher phenolic content, the fresh extract showed a higher antioxidant effect. MDA is a lipid peroxidation waste product with hazardous potential for normal homeostasis. Thiols, under oxidative stress, manage to form disulphide bonds between them to reduce oxidative stress. NO presents a dual effect based on its concentrations. Small doses possess an antioxidant effect, while high doses can cause an increase in oxidative stress through the synthesis of new and stronger radicals. 3NT is a waste product resulting from tyrosine nitration induced by reactive nitrogen species [94]. The authors concluded that GP extracts could be used considered a potential agent in nutraceuticals formulation.

An interesting study that evaluates the effects of red GP flour dietary inclusion on growth, anti-inflammatory, antioxidant, innate-adaptive immunity, and on immune genes expression was performed on *Labeo rohita* fish against *Flavobacterium columnaris* induced infection [95]. Treatment with 200 and 300 mg GP flour showed a significant increase in GSH, SOD, and GPx activities as compared to regular diet or 100 mg GP supplementation, in both infected and uninfected groups. Regarding GP action on innate-adaptive immune activity, higher doses of GP (200, 300 mg) increased phagocytosis, alternative-complement pathway activity, raised IgM levels, and serum lysozyme (Lyz) activity when compared to regular diet or 100 mg GP supplementation in infected or uninfected group. In terms of immune-related genes, Lyz, (β-2 microglobulin) β-2M, 3rd component complement (CC3), and immunoglobulin M (IgM) gene expression pointed out a significant growth in infected fish with 200, 300 mg GP supplementation compared to other groups. However, the uninfected group treated with the same doses of GP showed higher gene expression than the infected group. Antioxidant related-genes were measured, and SOD, GPx, nuclear factor erythroid 2-related factor 2 (Nrf2), and (natural killer-cell enhancing factor β) NKEF-β were remarkably higher in all groups treated with raised doses of GP compared to 100 mg GP diet or regular diet in infected or uninfected groups. Furthermore, uninfected groups treated with high doses of GP showed a more significant increase in SOD and GPx expression levels. As for pro-inflammatory-related genes, IL-1β and TNF-α were not modified in any group. Hepcidin and toll-like receptor-22 (TLR22) expression were increased in infected and uninfected groups treated with a high dose of GP [95].

Therefore, in Rajković et al. (2022), GP was given to piglets to assess their positive effects on the animal organism without antibiotics side effects [96]. During the experiment tissue samplings (liver, jejunum, ileum) were collected on days 27/28 and 55/56, while blood samples were taken on days 6, days 27/28, and 55/56. Regarding antioxidant enzymes, GPx (GPx1-liver, GPx-2 jejunum, and ileum) wasn’t different between diets, but the enzyme activity was significantly increased on days 55/56 compared to 27/28 [96]. About, SOD and Manganese Superoxide Dismutase (Mn-SOD) enzymes, there weren’t any differences between diets in jejunum, ileum, and liver, but there was an increase between sampling dates, in days 55/56 compared to 27/28 in the liver. The copper superoxide dismutase system (Cu-SOD or SOD1) presented no distinction between any sampling days in the liver or ileum. CAT activity wasn’t affected by any of the diets in the jejunum and liver, but there were differences between sampling days in the liver and ileum. TBARS concentrations weren’t affected by diets in any organs, only in the jejunum between sampling days (decreased levels on days 55/56 compared to 27/28). GPx2 and SOD1 gene expression were modified at the jejunum level (decreased in days 55/56 compared to 27/28), while CAT expression presented the same results at the ileum level. In the liver, the authors have observed differences between samples for SOD1, CAT, and GPx1 in the liver, with a higher expression on days 27/28 compared to 55/56. In terms of inflammation, pig major acute phase-protein serum levels presented a decrease on days 55/56 and 27/28 compared to day 6 [96]. MDA serum levels decreased through sampling days while for SOD different fluctuations were noticed, without showing any significant values on day 55/56 versus other time points. As a speculative explanation for the variation of antioxidant enzymes decreasing it can be stated that the systemic presence of antioxidant substances can lead to a decreasing need for endogenous antioxidant enzymes production [96]. 

Another important direction in GP research is to check whether it is suitable to be used as an adjuvant treatment in different pathologies to reduce conventional drugs side effects. Thus, in Mossa et al. (2015) study, cypermethrin was given to female rats to observe toxic effects on the liver and kidneys, and white GP was added to check whether it can counter these toxic effects [97]. The assessment of kidneys and liver biomarkers showed a dose-dependent fall in liver enzymes: aspartate transaminase (AST), alanine transaminase (ALT), gamma-glutamyl transferase (GGT), and alkaline phosphatase (ALP), and a decrease kidneys urea nitrogen and creatine. Also, total proteins and albumin revealed a significant increase in GP treated group. The histological analysis pointed out significant changes due to inflammatory infiltrate in cypermethrin groups, while the GP supplemented group had regressed values for all biomarkers. Similar results were also observed in histological studies of kidneys samples. This may be due to the antioxidant effects of the white GP [97]. This study offers important evidence regarding the use of GP extract with hepatorenal protective activity and encourages future studies to investigate whether it can be used to reduce other drugs adverse reactions. 

So far, the existing studies on GP suggest that through its anti-inflammatory and antioxidant effects, GP can be considered a potent agent that can contribute to the restoration of homeostasis to control levels or that can reduce different drug side effects (Table 2).

## 6. Non-Steroidal Anti-Inflammatory Drugs

Non-steroidal anti-inflammatory drugs (NSAIDs) represent a group of chemically distinct compounds that act through the same mechanism producing a reversible inhibition of cyclooxygenase (COX) 1 and 2 isoenzymes, enzymes that produce prostaglandins (PGs). The main representatives of the group of NSAIDs that act non-selectively on COX-1 and COX-2 are aspirin, ibuprofen, diclofenac, indomethacin, naproxen, ketorolac, piroxicam and meloxicam [119]. The main difference between these two isoenzymes is represented by the fact that while COX-1 is a constitutive enzyme which produce regularly PGs that have a protective role mainly on the stomach and kidney, COX-2 expression is induced by inflammatory stress and PGs resulted from this pathway lead to the swelling and pain associated with inflammation [120]. The ability of NSAIDs to reduce pain and swelling associated with several inflammatory diseases and the fact that NSAIDs are over-the-counter drugs, contributed to their large-scale use, being of the most sold drugs worldwide [121]. Besides these beneficial effects, NSAIDs have multiple adverse reactions, mainly related to COX-1 inhibition. Thus, the most common adverse reactions are the gastrointestinal ones. Long-term usage of NSAIDs could lead especially to gastric ulcer, but can also alter renal function and sodium exchange and could lead to hypertension and/or renal failure. On the cardiovascular system, COX inhibition could lead to heart failure exacerbation. On hepatic activity, COXs function alteration could lead to acute liver injury [121]. Hypersensitivity reactions are also described and manifest clinically by NSAIDs-exacerbated respiratory diseases (rhinosinusitis, bronchial asthma, pneumonitis), NSAIDs-exacerbated cutaneous diseases (urticaria, photo-contact dermatitis, angioedema), NSAIDs-induced diseases (nephritis, aseptic meningitis) and anaphylaxis [122,123,124,125]. Because of these multiple and heterogenous adverse effects associated with COX-1 inhibition, pharmacology researchers discovered other compounds from the class of NSAIDs, celecoxib, which selectively inhibits COX-2. However, although studies have shown a reduced adverse effect on the gastrointestinal system, namely a lower risk of gastric ulcer, this compound more frequently leads to acute coronary syndromes [121]. Besides the anti-inflammatory activity of NSAIDs, another area of interest was represented by their antioxidant activity. In this way, Costa et al. (2006) focused on the antioxidant activity via the *in vitro* scavenging ability of ROS and reactive nitrogen species (RNS) of ibuprofen, flurbiprofen, fenbufren, fenoprofen, naproxen, ketoprofen and indoprofen [126]. They observed that the greatest scavenging activity of ROS for O^2−^ was equal for fenbufen, flurbiprofen, indoprofen, ketoprofen, for H_2_O_2_ was equal for ketoprofen, indoprofen, fenbufen and for HO was equal for fenoprofen and ibuprofen. In the case of RNS, for NO the greatest scavenging activity was that of indoprofen, and for ONOO^−^ was indoprofen [126].

However, in the *in vivo* studies no antioxidant activity was observed, but, on the contrary, a prooxidant effect was described. One example is represented by the study of Nawaz et al. (2021) who observed the effect of NSAIDs treatment on antioxidant status and oxidative stress in patients with rheumatoid arthritis [127]. They highlighted that the group of patients with rheumatoid arthritis under the treatment with NSAIDs, compared with the other three groups (control group, patients without rheumatoid arthritis and under the treatment with NSAIDs, patients with rheumatoid arthritis who did not take NSAIDs), showed the highest oxidative stress and the lowest free radical scavenging ability [127].

Taking into consideration all of the above, namely the multitude of adverse reactions associated with NSAIDs treatment, it is necessary to find a potent substitute for these drugs, one that has strong anti-inflammatory effects with as few adverse reactions as possible.

## 7. Comparative Effect of Polyphenols Found in Grape Pomace Versus Non-Steroidal Anti-Inflammatory Drugs in Oxidative Stress and Inflammation 

Several diseases use NSAIDs to decrease vasodilatation, cell adhesion to vascular endothelium, cells migration to inflammation site, cytokine synthesis, and further tissue damage. However, as we already mention before, NSAIDs also possess several adverse reactions (gastrointestinal bleeding, hepatic, renal toxicity), which can cause a limitation in use for more extended periods. This led to studying different natural compounds to compare their effects with different NSAIDs (Table 3).

Unfortunately, we were unable to find studies in which the anti-inflammatory and antioxidant effects of GP whole extract were compared with those induced by NSAIDs, but only of several other compounds extracted from GP, such as resveratrol, flavonol (quercetin) and hydroxybenzoic acid (gallic acid). Therefore, this section will examine the differences between *in vitro* and *in vivo* effects of NSAIDs and different phenolic compounds that are also found in GP.

Thus, Zhang et al. (2021), subjected rat glial cells to LPS induced-inflammation and treated them with different doses of resveratrol (0.1–20 µM) or ibuprofen to see the differences [136]. Resveratrol showed a higher decrease in TNF-α (0.1–20 µM) and ROS production (20 µM) compared to ibuprofen [136]. In another study, horse immune cells were cultured with resveratrol or different NSAIDs (flunixin meglumine or phenylbutazone) to observe the effects on pro-inflammatory cytokine production. Resveratrol managed to lower interferon (IFN)-ɣ and TNF-α levels, similar to of NSAIDs [137].

As for *in vivo* studies, resveratrol patches were given to rats injected with carrageenan [138]. Diclofenac gel was used to compare the effects of the two substances. Resveratrol and diclofenac decreased paw swelling, but resveratrol caused a much longer inflammation reduction when compared to diclofenac. Also, the resveratrol group presented the lowest paw swelling compared to the diclofenac and control groups [138].

Another polyphenol found in GP and recognized for its anti-inflammatory and antioxidant effects is represented by quercetin, which is a flavonol. In Wei et al. study (2019), osteoarthritis was surgically induced in the left knee in rats, that were also treated with quercetin or celecoxib (CXB) as a reference drug [139]. In terms of antioxidant effects, quercetin and CXB groups managed to increase SOD serum and synovial fluid levels compared to the untreated group. Metalloproteinases (MMPs) are a family of enzymes responsible for cartilage degradation and osteoarthritis appearance [139]. Tissue inhibitor metalloproteinase (TIMP) is accountable for cartilage repair through the downregulation of MMPs [139]. In this study, quercetin and CXB groups decreased MMP-13 in serum, synovial fluid, and synovium, while TIMP-1 levels increased in these groups compared to the untreated osteoarthritis group [139]. In this work, quercetin presented comparable effects with celecoxib, both having protective effects [139]. Heydari Nasrabadi et al. (2022) also analyzed the effects of quercetin in monosodium iodoacetate-induced osteoarthritis in rats injection [140]. In this experiment, ibuprofen was used as a reference drug. In histopathological studies of the rats’ knees, the inflammatory cells were significantly decreased in the treated groups (quercetin and ibuprofen) compared to the untreated group. In addition, MMP-3 and MMP-13 were measured, and the results showed that the quercetin group reduced, even more, the levels of MMPs compared to the ibuprofen group [140].

When taking into consideration the oxidative stress induced by the treatment with NSAIDs, other researchers focused on finding a substance that could limit this pro-oxidative side effect. In this matter, a great phenolic compound found in high quantities in GP, gallic acid, has proven to be a potent adjuvant in this case. Thereby, Moradi et al. (2021) evaluated if gallic acids taken alongside diclofenac had an impact on the oxidative status of 30 Wistar rats divided into 5 groups [141]. They observed that while only under diclofenac treatment there was a significant increase in urea, AST, ALT, creatinine, uric acid serum levels, and in IL-1β expression, in the group which received gallic acid after diclofenac there were registered reduced levels of these biochemical parameters and gene expression [141]. Moreover, the treatment with gallic acid alongside diclofenac led to a decrease in nitrate content, serum and renal MDA levels, and protein carbonyl level. Furthermore, on antioxidant enzymes activity, treatment with diclofenac and gallic acid led to an increase in renal SOD and CAT activities and renal GSH levels. The histopathological examination of the kidney highlighted that the group which received both diclofenac and gallic acid presented a reduced lymphocytic cell infiltration, focal hemorrhage, and vacuolar degeneration of tubular epithelial cells in comparison with the group which received only diclofenac [141]. Another study that reinforces these observations is that of Esmaeilzadeh et al. (2020) who investigated the hepatoprotective activity of gallic acid in diclofenac-induced liver toxicity in 30 Wistar rats [142]. They observed that while diclofenac led to an increase in GOT, GPT, ALP, and total bilirubin levels, gallic acid reduced the levels of these parameters. Moreover, at a dose of 100 mg/kg gallic acid, there was observed a significantly decreased compared with a dose of 50 mg/kg gallic acid [142]. The same difference between these two doses was also observed in the increase of plasma antioxidant capacity, hepatic SOD, GPx, GSH, and CAT activities and in the decrease of nitrite content, protein carbonyl levels, serum, and liver MDA levels compared with the group which received only diclofenac. The histopathological examination of the liver, both doses of gallic acid led to a significantly reduced in lymphocytic cell infiltration and liver degeneration [142].

These *in vivo* studies also suggest that GP is a valuable candidate that could be used as a potential therapeutic agent capable of reducing oxidative stress and inflammation and also as an adjuvant treatment in the attempt to reduce the side effects.

## 8. Conclusions

GP extracts due to their rich content in flavonols, anthocyanins, anthocyanidins, flavanols, proanthocyanidins, stilbenes, and phenolic acids are offering new perspectives as possible therapeutic agents. In vivo and *in vitro* studies showed promising results for both GP whole extracts and different types of polyphenols that are contained in it, in reducing oxidative stress and inflammatory markers in different models of chronic inflammation. 

The first evidence in the direction of this possible therapeutic use was represented by in vitro studies. In these works, the antioxidant effects were demonstrated especially by the decrease in ROS, MDA, and TBARS levels and by the increase in GSH levels. The anti-inflammatory effects were given by the inhibition of some inflammatory pathways such as NF-kB and PGE2, an inhibition that led to a decrease in levels of inflammatory markers such as IL-8. These antioxidant and anti-inflammatory effects are also validated in *in vivo* studies. In terms of antioxidant activity, in addition to the effects already observed in the *in vitro* studies, an increase in CAT, SOD, and GPx4 levels and stimulation of eNOS gene expression were also seen. Similarly, the *in vivo* studies brought additional data regarding the anti-inflammatory activity, observing an inhibition of the release of several inflammatory markers such as IL-1𝛼, IL-1β, IL-6, IFN-𝛾, TNF-α, and CRP. Anyway, it is still necessary to implement this research in clinical trials to be able to conclude the possible use of GP as an antioxidant and anti-inflammatory therapeutical agent. Further, most of the studies have shown that GP extracts are more effective than a single polyphenol, possibly because of polyphenols synergistic action that interferes with more than one pathophysiological mechanism. Compared to the antioxidant and anti-inflammatory effects of NSAIDs, the literature currently offers only the effects of single polyphenols, such as resveratrol, quercetin, and gallic acid. However, single polyphenols possess similar anti-inflammatory and antioxidant effects as the classical NSAIDs. Thereby, further comparative studies are needed to investigate if the entire complex of polyphenols that exists in GP may induce a similar or a better effect. The question that rises from these studies is whether the best results can be obtained when single isolated phenolic compounds with a known concentration are used or whether a standardized mixture of these compounds is used? If this hypothesis is validated, in the future GP could become an add-on therapeutic measure that could be used for better control of chronic inflammation than monotherapy with NSAIDs.

## Figures and Tables

**Table 1:** *In vitro* beneficial actions of grape pomace in oxidative stress and inflammation.

Grape Pomace Variety	Models	Polyphenols Content	Antioxidant and Anti-Inflammatory Effects	References
***Red Grape Pomace Variety***				
Batiki Tyrnavou variety (Greece)	Tert-butyl hydroperoxide induced-oxidative stress in C2C12 muscle cells and EA.hy926 endothelial cells	Flavanols, (catechin and epicatechin), anthocyanidins, anthocyanins, flavonols (quercetin) phenolic acids (gallic acid and caftaric acid)	-decreased ROS levels in muscle cells -decreased TBARS and carbonyls levels in both cells line -increased GSH levels in both cells line;	[84]
Tinta Cao and Cabernet Franc (USA)	CA77 cell line	-	-decreased CGRP levels	[85]
Tempranillo variety (University of Burgos, Spain) -WGPI-gastrointestinal digestion; WPF—colonic fermentation	Hyperglycemic treatment in EA.hy926 endothelial cells	Phenolic acids, flavanols, stillbenes, flavonols	-increased mRNA Nrf2 levels, pNrf2/Nrf2 ratio; -increased pAkt/Akt ratio; -decreased pIκBα/IκBα and pIKK/IKK ratio, mRNA COX2, NOX4, SOD2 levels; -increased mRNA SIRT1, HO-1, CAT, NQO1 levels, GSH/GSSG ratio; -increased mRNA GS, GR levels in WGPI; -increased phospho-p38-MAPK/p38-MAPK ratio in WPF; -decreased mRNA NF-κB levels and pNF-κB p65/NF-κB p65 ratio in WPF; -increased mRNA GCLC, GS, GR, SOD, GPx1 levels in WPF;	[86]
Carignan variety (Northern Tunisia)	-6-hydroxydopamine-induced oxidative stress in mesencephalic cells (dopaminergic cells) -6-hydroxydopamine-induced oxidative stress in dopaminergic cells derived from stem cells	-	-increased cell viability in mesencephalic primary cells; -decreased ROS production in stem cells; -decreased phospho-NF-κB p65 translocation	[87]
***White grape pomace variety***				
Batiki Tyrnavou variety (Central Greece)	Bovine spermatozoa incubated with different GP concentrations	-	-decreased MDA levels;	[88]
***Red and White pomace variety***				
White grape pomace (Moscato branco) and mixed grape pomace (red + white) Enzymatic hydrolysis treated fractions	IL-1β treated Caco-2 cells	Quercetin, catechin, resveratrol, gallic and caffeic acids, trans-resveratrol, rutin and procyanidin B2	-decreased ROS in all fractions (100-200 µg/mL); -decreased NF-κB, PGE2 levels in all fractions; -significantly greater decrease of IL-8 levels in mixed grape pomace with or without enzymatic hydrolysis;	[89]

Abbreviations: CAT—catalase; CGRP—calcitonin gene-related peptide; COX 2—cyclooxygenase 2; GR—glutathione reductase; GS—glutathione syntase; GCLC—glutamate-cysteine ligase catalytic subunit; GPx1—glutathione peroxidase 1; GSH—glutathione; GSSG—glutathione disulfide; HO-1—heme oxygenase 1; MDA—malondialdehyde; NF-κB—nuclear factor kappa-light-chain-enhancer of activated B cells; NF-κB p65—nuclear factor kappa-light-chain-enhancer of activated B cells transcription factor; NOX4—nicotinamide adenine dinucleotide phosphate oxidase 4; NQO1 —nicotinamide adenine dinucleotide plus hydrogen quinone oxidoreductase 1 mRNA Nrf2—messenger ribonucleic acid nuclear factor erythroid 2-related factor 2; p38-MAPK—p38mitogen-activated protein kinase PGE2—prostaglandin E2; pAkt—phosphorylated protein kinase B; pIκBα—phosphorylated inhibitor of kappa B; pIKK—phosphorylated IκB kinase; pNrf2—phosphorylated Nrf2 ROS—reactive oxygen species; SIRT1—sirtuin 1; SOD2—superoxide dismutase 2; TBARS—thiobarbituric acid reactive substances; TNF-α—tumor necrosis factor alpha.

**Table 2:** *In vivo* beneficial actions of grape pomace in oxidative stress and inflammation.

Grape Pomace	Models	Polyphenols Content	Antioxidant and Anti-Inflammatory Effects	References
***Red grape pomace variety***				
Tempranillo variety (Burgos, Spain)	Spontaneously hypertensive rats	Proanthocyanidin, anthocyanins, quercetin	-TAC increased; -decreased lipid peroxidation and carbonyl groups; -increased NO levels -increased HO-1, SOD2, eNOS gene expression;	[98]
Alicante and Pinot varieties (France) polyphenol-enriched Alicante	Dextran Sulfate Sodium-Induced colitis in Wistar male	Anthocyanins	-improved histological score; -decreased MPO activity; -increased SOD activity in polyphenol-enriched Alicante; -decreased IL-1𝛼, IL-6 IFN-𝛾 levels; -decreased IL-1β levels in Alicante and Pinot; -decreased IL6, ICAM-1, MMP-9 gene expression; -decreased IL-1β, iNOS gene expression in Alicante and Pinot; -decreased TNF𝛼, NF𝜅B p65, COX2 gene expression in polyphenol-enriched Alicante;	[99]
Malbec variety (Gualtallary, Mendoza, Argentina)	High fructose diet-induced Metabolic syndrome in Wistar rats	Quercetin, epicatechin, catechin, trans-resveratrol, ferulic, gallic, caffeic, syringic, p-coumaric acids	-reduced CRP levels; -reduced NADPH oxidase activity; -increased adiponectin; -reduced resistin; -increased insulin sensitivity;	[100]
Dimrit grapes variety	96 laying Hens given different GP concentrations	Catechin, Epicatechin, Gallocatechin, Epigallocatechin, Phenolic acids, Gallic acid, Caffeic acid, p-cumaric acid	-decreased plasma MDA levels; -decreased egg yolk MDA levels;	[101]
Red wine grape pomace	18 crossbreed lambs given different GP diets (5%, 10%)	-	-increased TAOC and SOD, GPX activity in longissimus dorsi muscle; -decreased ROS and MDA levels in longissimus dorsi muscle;	[102]
Red wine grape pomace	24 crossbreed ram lambs under pen conditions given GP diets (5%, 10%)	-	-decreased MDA and ROS levels in lamb testes; -increased CAT, SOD, GPx4 activity in lamb testes; -increased TAOC (GP 10%); -increased SOD, GPx4 mRNA expression (GP 10%); -increased CAT, SOD, GPx4 protein abundance;	[103]
Tempranillo variety	Wistar rats given high-fat diet	-	-decreased IL-1β and TNF-α levels; -increased FRAP plasma and liver levels; -increased liver GSH/GSSG ratio; -decreased plasma and liver MDA and carbonyl groups levels; -decreased 8-hydroxydeoxyguanosine plasma levels;	[104]
Muscat Bailey A variety (Gyeongsangbuk-do, Korea)	High-fat diet induced obesity in male C57BL/6J mice	Catechins, resveratrol, flavonoids,	-decreased TNF-α, PAI-1 levels; -decreased liver NF-κB, IL-6 and TNF-α levels;	[105]
Cabernet Franc (Chrysalis Vineyards, Virginia)	C57BL/6NCr mice given high-fat diet	-	-decreased TNF-α, IFN-ɣ, IL-12β, PAI-1 and resistin levels	[106]
Red wine pomace	78 crossbreed piglet given apple or grape pomace	Flavanols	-decreased NF-κB mRNA expression in the stomach; -increased NF-κB and TNF-α mRNA expression in the liver and muscle; -decreased TNF-α and IL-10 mRNA expression in ileum; -increased IL-10 mRNA expression in the jejunum, colon, and liver;	[107]
Red dried grape pomace (*Vitis vinifera* L. variety)	20 Fresian cows given GP diet	Flavonoids, gallic acid, epicatechin	-lower MDA levels in the cheese from cow’s milk that received GP; -lower thrombogenic index in cow’s milk that received GP;	[108]
Pinotage variety (Bellevue Wine Estate, Stellenbosch, South Africa)	40 lambs given GP diets at different c% (0, 5, 10, 15, 20)	Proanthocyanidins, tannins	-increased antioxidant activity (15, 20% diets) within first 3 days of meat storage; -decreased TBARS levels of all diets from day 5 to day 9 of meat storage; -decreased carbonyl content in stored meat (10, 20%);	[109]
Pinotage variety (Bellevue, Beyers Kloof, Western Cape Province, South Africa)	Angus steer given dried grape pomace or dried citrus pulp	Proanthocyanidins, tannins	-decreased TBARS and carbonyl levels; -increased antioxidant activity;	[110]
Cencibel variety (Grupo Matarromera San Bernardo-Valbuena de Duero, Valladolid, Spain) Enzymatic hydrolysis treated fractions—tannase and carbohydrase enzyme complex—separately or combined	300 Cobb chicks given different GP c% (5, 10) diets—hydrolyzed/un-hydrolyzed	Gallic acid, Catechin, Epicatechin, Procyanidin B1, Procyanidin B2 Epicatechin-O-gallate;	-decreased MDA levels;	[111]
Moschato variety Tyrnavos (Larissa prefecture, Greece)	30 female broilers given GP diet for 15 or 35 days;	-	-15 days GP diet: decreased TBARS plasma levels, increased GSH levels in kidney and spleen, decreased TBARS levels in pancreas and intestine, decreased CARB levels in the kidney; -35 days GP diet: increased GSH erythrocytes levels, decreased TBARS plasma levels, increased GSH levels in kidney, spleen, heart, lung, and liver, increased TAC levels in liver, spleen, and kidney, decreased H_2_O_2_ decomposition in the intestine, decreased TBARS levels in spleen, quadriceps muscle, and heart, decreased CARB levels in spleen and kidney;	[112]
Cencibel variety	180 broiler chicks given different GP diets doses (15, 30, 60 mg/kg) or Vitamin E;	Condensed tannins	-decreased MDA levels in refrigerated breast meat;	[113]
Cencibel variety (Vinícola de Castilla S.A.,Manzanares, Ciudad Real, Spain)	120 broiler chicks given different GP diets doses (5, 15, 30 mg/kg) or Vitamin E;	-	-decreased MDA levels in refrigerated breast and thigh meat (day 7); -decreased MDA levels in refrigerated breast meat (day 4);	[114]
Moschato variety (Tyrnavos Larissa, Greece)	24 piglets given GP diet (blood and tissue samples taken at 15 and 30 days post-diet)	-	-15 days GP diet: decreased TAC plasma activity, decreased CARB levels in spleen, brain and liver, decreased TBARS levels in brain, kidneys, stomach, heart, lungs, quadriceps muscle, and spleen, increased TAC levels in stomach and pancreas, decreased TAC levels in the brain, increased GSH levels, heart, liver, spleen, stomach, pancreas, lungs, brain and quadriceps muscle; increased H_2_O_2_ decomposition activity in kidneys and decreased in lungs and stomach; -30 days GP diet: decreased CAT erythrocytes activity, decreased CARB levels in spleen, brain, liver, lungs, quadriceps muscle, stomach, and pancreas, decreased TBARS levels in brain, liver, heart, lungs, quadriceps muscle, spleen, and pancreas; increased TAC levels in the quadriceps muscle, kidneys, lungs, stomach, and pancreas, decreased TAC levelsthe in brain, increased GSH levels heart, liver, pancreas, lungs, brain and quadriceps muscle, kidneys; decreased TAC levels in stomach and spleen; increased H_2_O_2_ decomposition activity in kidneys, quadriceps muscle, pancreas and decreased in lungs and brain;	[115]
Cencibel variety (La Mancha, España)	70 broiler chicks given GP diets doses (0, 30, 60 mg/kg)	Condensed tannins, hydrolysable tannins;	-reduced TBARS levels in raw chicken patties (storage day 13, 20); - reduced TBARS levels in cooked chicken patties (storage day 3, 6, 13, 20); -reduced TBARS levels in raw chicken patties (60 mg/kg—6 months storage); -reduced TBARS levels in cooked chicken patties (30, 60 mg/kg—6 months storage);	[116]
Moschato variety (Tyrnavos Larissa, Greece)	28 lambs given GP diet (blood and tissue samples taken at 27 and 55 days post-diet)	-	-27 days GP diet: increased CAT erythrocytes activity; decreased protein carbonyls level in the liver; increased TBARS activity in the brain; -55 days GP diet: reduced TBARS activity in liver, spleen, and heart; increased TBARS activity in the brain; decreased TAC in brain and liver; GSH levels increased in quadriceps muscle and spleen; decreased GSH levels in the liver;	[117]
Carignan variety (Northern Tunisia	Adult mice given 6-hydroxydopamine stereotaxic injection in midbrain (Parkinson disease model)	-	-increased SOD1 brain levels; -decreased neurons depletion in substantia nigra; -ameliorated motor impairment;	[92]
***White grape pomace variety***				
Koshu variety (Japan and Italy) Fermented or un-fermented fractions	Female rats induced-allergic reactions (asthma and passive cutaneous anaphylaxis)	-	-decreased serum IgE levels; -decreased eosinophils levels in bronchial lavage; -decreased cutaneous reaction in time and dose-dependent manners; -decreased cutaneous reaction compared to Tannat or Negroamaro GP (red varieties)	[118]

Abbreviations: AOPP—advanced oxidation protein product; CARB—protein carbonyls; CAT—catalase; COX 2—cyclooxygenase 2; CRP—C-reactive protein; DPPH—2,2-diphenyl-1-picrylhydrazyl; FRAP—ferric ion antioxidant reducing power; GP—grape pomace; GPx—glutathione peroxidase; ɣ-GCS—ɣ-synthase glutamyl cysteine; GSH—glutathione; GSSG—glutathione disulfide; GST—glutathione-s-transferase; HO-1—heme oxygenase 1; ICAM-1—Intercellular Adhesion Molecule 1; IFN-ɣ—interferon gamma; MDA—malondialdehyde; MMP-9—matrix metalloproteinase 9; MPO—myeloperoxidase activity; NADPH—nicotinamide adenine dinucleotide phosphate; NF-κB p65—nuclear factor kappa-light-chain-enhancer of activated B cells transcription factor; eNOS- endothelial nitric oxide synthase; iNOS—inducible nitric oxide synthase; NO—nitric oxide; oxLDL- Oxidized low-density lipoprotein; PAI-1—Plasminogen activator inhibitor-1; ROS—reactive oxygen species; SOD—superoxide dismutase; TAC—total antioxidant capacity; TAOC—total antioxidant capacity; TAS—total antioxidant status; TBARS—thiobarbituric acid reactive substances; TNF-α—tumor necrosis factor alpha.

**Table 3:** Grape pomace polyphenols versus non-steroidal anti-inflammatory drugs in oxidative stress and inflammation.

Administrated Polyphenol	NSAID	Model	Anti-Inflammatory and Antioxidant Effects of Polyphenols	Differences of Results Between Polyphenols and AINS	References
RES 50 mg/kg	Diclofenac 3 mg/kg	Adjuvant-induced arthritis in rats	-reduced TNF-α, IL-1β, TBARS and NOx levels; -reduced NF-κB p65 expression;	-	[128]
RES 50 mg/kg	Diclofenac 3 mg/kg	Adjuvant-induced arthritis in rats	-reduced TNF-α, IL-1β, TBARS and NOx levels; -attenuated histological changes (cartilage damage, pannus formation, cellular infiltration, synovial proliferation) -reduced NF-κB p65 expression;	-diclofenac group decreased paw volume	[129]
RES 10/50 mg/kg	Celecoxib 5 mg/kg	Adjuvant-induced arthritis in rats	-reduced paw volume; -decreased lymphocyte proliferation; -decreased COX2 expression; -decreased PGE2 levels;	-resveratrol exhibited similar results to celecoxib	[130]
RES 100 mg/kg	Celecoxib	Lipopolysaccharide induced-sepsis in rats	-no significant reduction of prostaglandin plasma and kidneys levels; -no significant reduction of mRNA MCP-1 and IL-6 levels;	-celecoxib tackle the inflammation effects compared to resveratrol	[131]
RES 10 mg/kg	Ibuprofen 30 mg/kg	Experimental arthritis and periodontitis in rats	-longer reduction in paw swelling; -higher gingival IL-4 levels;	-	[132]
RES 5/10 mg/kg	Etoricoxib 10 mg/kg	Experimental osteoarthritis induced in rats	-increased time of paw withdraw mechanical, heat and cold hyperalgesia test in RES groups; -increased spontaneous rats’ movement in RES groups; -decreased serum TNF-α, IL-10 levels; -decreased serum IL-1β levels (RES 10 mg/kg); -decreased synovial TNF-α, IL-10 and IL-1β levels; -decreased cartilage mRNA TNF-α, IL-10 expression; -decreased cartilage mRNA IL-1β expression (RES 10 mg/kg); -decreased cartilage protein TNF-α, IL-10, IL-1β, IL-6, MMP-13 expression; -decreased mRNA iNOS expression; -decreased mRNA COX2 expression (RES 10 mg/kg); -decreased protein iNOS, COX2 expression;	-etoricoxib manage to present same anti-inflammatory effects as RES 10 mg/kg treated group;	[133]
Quercetin (75 mg/kg)	Phenylbutazone (80 mg/kg) and Indomethacin (6 mg/kg)	Freund’s complete adjuvant-induced arthritis; carrageenan-induced paw edema	-reduced paw edema (carrageenan experiment); -reduced paw volume in the acute phase; -reduced paw volume in the chronic phase (days 8, 9, 10, 14, 15, 16)	-higher anti-inflammatory effect compared to phenylbutazone in carrageenan experiment;	[134]
Quercetin (80 mg/kg)	Phenylbutazone (80 mg/kg)	Experimental induced-arthritis in rats	-reduced paw volume in the acute phase; -reduced paw volume in the chronic phase (day 9, 10, 14, 16, 19, 23, 26 and 30)	-	[135]

Abbreviations: COX2—cyclooxygenase 2; iNOS—inducible nitric oxide synthase; MCP-1—monocyte chemoattractant protein-1; MMP-13—mettaloproteinase 13; NF-κB p65—nuclear factor kappa-light-chain-enhancer of activated B cells transcription factor; NOx—serum total nitrate/nitrite; PGE2—prostaglandin E2; RES—resveratrol; TBARS—thiobarbituric acid reactive substances; TNF-α—tumor necrosis factor alpha.

## Data Availability

Not applicable.
